# Demographics and practices of semi-intensive free-range farming systems in Australia with an outdoor stocking density of ≤1500 hens/hectare

**DOI:** 10.1371/journal.pone.0187057

**Published:** 2017-10-24

**Authors:** Mini Singh, Isabelle Ruhnke, Carolyn de Koning, Kelly Drake, Alan G. Skerman, Geoff N. Hinch, Philip C. Glatz

**Affiliations:** 1 Sydney School of Veterinary Science, University of Sydney, Camden, New South Wales, Australia; 2 Animal Science, School of Environmental and Rural Science, University of New England, Armidale, New South Wales, Australia; 3 South Australian Research and Development Institute, Roseworthy, South Australia, Australia; 4 Department of Agriculture and Fisheries, Toowoomba, Queensland, Australia; Gaziosmanpasa University, TURKEY

## Abstract

Baseline information on demographics and practices on semi-intensive free-range egg farms with an outdoor stocking density of ≤1500 hens/hectare in Australia is presented. Free-range egg production is changing the structure of the egg industry in Australia and a broad variety and tiers of free-range systems have emerged due to lack of concrete legislative standards on outdoor stocking densities in the past. Information was extracted from a pre-existing online free-range poultry survey dataset, consisting of a total of 79 questions related to nutrition, pasture management, welfare and health, animal housing, environmental impact and economics. Forty-one free-range egg farms, with an outdoor stocking density of ≤1500 hens/hectare, were identified in the dataset from all major Australian states. Two types of semi-intensive free-range housing systems were documented: mobile (modified caravan/trailer) housing (56%), and fixed sheds (44%). Seventy-two percent of respondents reported >75% of the hens in the flock used the outdoor range. All respondents reported ingestion of range components by hens in the form of vegetation, insects, stones and grit. Up to 10% mortality was reported by 40% respondents with predation (34%), cannibalism (29%), heat stress (24%) and grass impaction (19.5%) as major causes. Biosecurity on farms was sub-optimal with 8 of the 10 actions implemented by <50% respondents. Customer demand, consumer sentiment and welfare were the major factors for farmers moving into free-range egg production. This study resulted in identification of current practices and key challenges on semi-intensive free-range egg farms. Applied research and communication of results to farmers is highly recommended to ensure optimum health and welfare of free-range laying hens and sustained egg production.

## Introduction

Recent legislation in Australia, that came into effect from March 2017, states that eggs labelled ‘free-range’ need to be laid by hens with meaningful and regular access to the outdoors and that there would be a ceiling on outdoor stocking density of 10000 hens/hectare [1]. However, earlier model Code of Practice [2] and standards from animal welfare bodies [3, 4], as well as many established semi-intensive free-range egg farmers, have often advocated the maximum stocking density for the outdoor area as ≤1500 hens/hectare. The lack of concrete legislative standards on outdoor stocking densities in the past has resulted in the emergence of a wide variety and tiers of free-range farms in Australia as compared to elsewhere in the world. Intensive free-range systems typically consist of fixed sheds with pop-holes opening on sides of sheds leading to either fixed or rotational range areas with an outdoor stocking density of up to 10,000 birds/ hectare and are mostly affiliated to industry service bodies. However, there are a number of producers that run their egg layers in semi-intensive systems, some consisting of mobile units that can be moved regularly to fresh pasture. In a number of these farms, hens are free to roam large range areas usually with outdoor stocking densities of ≤1500 hens/hectare.

Free-range egg production is a rapidly growing sector in Australia with an estimated grocery market value share of 50.6% of the total grocery egg sales value of AUD $880.8 m (~US $658 m) for the 2015/16 financial year [5]. This is in line with the expansion of the free-range sector worldwide. As of September 2016, organic and cage-free shell egg production accounted for 12.5% of current table egg layer flock (37.6 million hens) in USA [6]. In a latest report from UK, free-range eggs accounted for 48% of total throughput in the first quarter of 2017, three percentage points higher than the previous year [7].

Although the majority of free-range eggs are produced on intensive free-range farms, meeting retailer and consumer demands remains a challenge for the poultry industry in Australia. In order to fill this gap semi-intensive free-range farmers are emerging, that sell eggs at the farm gate, farmers’ markets or to regional stores [8]. One of the reasons for the upward trajectory in demand for poultry products produced in less intensive production systems comes from the perception that these systems are superior for bird welfare, product quality and food safety [9]. A market research report for the Australian Egg Corporation Limited, looking at consumer’s understanding of egg production methods revealed the general perception that free-range and organic made for “a better quality egg” [10]. In a survey on consumer perceptions of free–range laying hen welfare in UK, consumers perceived hens in this system to be ‘happier’ and ‘healthier’, and believed that the free-range eggs ‘tasted better’. Moreover, ‘outside access’ and ‘fresh air’ were considered to be the most important factors contributing to hen welfare in this study [11]. In Belgium, the most important reason for farmers to rear their birds in a free-range system, after the ban on conventional cages in the EU, was based on consumer demand [12]. A study looking at modelling purchaser attributes and demographics in Canada found that non-caged egg consumers were less concerned about price, had higher awareness about different types of table eggs, purchased their eggs from local/organic grocery stores, farm gates or farmers markets, and were more concerned about care and feeding of hens compared to consumers of other eggs type [13].

Much of the information on poultry management practices available to date are based on research conducted in conventional intensive cage systems. Farmers who venture into free-range systems do not have the advantage of well-documented and researched practices. Many farmers have adapted and modified existing infrastructure and methodologies to suit their specific farm’s requirements. Our current knowledge base is limited on semi-intensive free-range egg production methods and how this production system affects hen performance and egg quality.

Free-range egg production has been associated with a number of challenges including production gap, nutrient dilution from pasture consumption, range management, biosecurity and losses due to predation, cannibalism, grass impaction, parasites and disease [14–17]. Education and training are required to achieve dissemination of sound scientific knowledge to the farmers. The level of adoption of free-range systems and its long-term sustainability also needs to be developed.

In order to address the above issues, baseline information on semi-intensive free-range farms in Australia with an outdoor stocking density of ≤ 1500 hens/hectare has been documented for the first time in this study. Data was extracted from a pre-existing free-range poultry survey for farm and flock characteristics, housing, outdoor range and its components, range access, production, health, welfare, biosecurity, environmental impact and factors influencing adoption and sustainability of semi-intensive free-range farms. Information on these farms is essential to fully understand the scope of free-range egg production in Australia.

## Materials and methods

### Dataset

For this study, a subset of non-identifiable data was selected from a pre-existing dataset that was generated by the authors for the Australian Poultry Co-operative Research Centre through an online questionnaire on the internet survey host SurveyMonkey^®^ (http://www.SurveyMonkey.com) and sought participation from free-range poultry farmers across Australia. The survey questionnaire and participant information sheet (PIS) used in the study were prepared under the ethical guidelines of the Australian Poultry Co-operative Research Centre. Respondents in the original survey were informed about the purpose of the survey, future use of information and the privacy policy on the introduction page of the online survey. They were required to provide an electronic consent, where clicking on the "agree" button indicated that they had read the information on the page and that they voluntarily agreed to participate in the study. If they did not wish to participate in the research study, they could decline participation by clicking on the "disagree" button. The design of the survey prevented respondents being directed to the questionnaire if they did not give their consent.

The original dataset was created by analysing and exporting data from SurveyMonkey^®^ to an Excel spreadsheet and stored after de-identification of respondents. The questionnaire comprising of 79 questions was developed with careful consideration given to construct, content, and the ability of this platform to measure and collect relevant information. SurveyMonkey^®^ provided the option to transfer survey responses directly into a database, eliminating transcription errors and preventing alteration by the survey respondent.

### Sampling frame

A specific subset of data was selected, with the sampling frame designed to include participants only if they were: 1) a semi-intensive free-range egg producing farm; 2) with an outdoor stocking density of ≤1500 hens/hectare; and, 3) had answered all 79 questions in the original dataset. These selection criteria were used to gain information on a segment of the industry that has been under-reported in the literature with limited information on their contribution to the total egg production. The main categories in the data subset for this study and information recorded for each of these categories is outlined in Table 1 and the minimal dataset presented as supporting information file (S1 Dataset).

**Table 1:** Main categories and information within each category recorded in the survey questionnaire.

Main categories	Information recorded
**1. Participants information**	address, phone number, e-mail and website information
**2. Farm and flock characteristics**	number of farms owned, breed of hen, number of flocks housed per year, average flock size, total number of hens/farm, the range and its components, range access, climatic conditions
**3. Housing and feeding**	climate control, feeders and drinkers, feed characteristics, nesting
**4. Rearing and production**	pullet rearing, laying hen performance
**5. Health and welfare**	mortality rate, cause of mortality, most prevalent disease, parasites, frequency of veterinarian visits
**6. Biosecurity**	biosecurity measures practiced on farm
**7. Manure management**	vegetative cover and water bodies, manure and surface run-off management
**8. Factors influencing free-range farming**	reasons for adoption of this type of farming, anticipated years in operation, support system, skill base, access to scientific knowledge, areas priortised for research.

### Statistical analysis

The levels of the different variables, along with the associated frequency of occurrence and probabilities were calculated using JMP^®^, Version *11*.*2*.*0*. (SAS Institute Inc., Cary, NC, USA), which included Count (the number of occurrences found for each level of a response), Response (%) (the probability (proportion) of occurrence for each level of a response variable, where the probability computed by dividing the Count by the total occurrences of the variable), CI (95% lower and upper confidence intervals computed using the score confidence intervals) and SE (Prob) (the standard error of the probability). Where more than one option was chosen for an answer in a multiple-choice question, probability percentages were obtained for each option separately. For ranking questions, average ranking for each answer choice was weighted in order to determine which answer choice was most preferred overall. Weights were applied in reverse, where respondent's most preferred choice had the largest weight, and their least preferred choice had a weight of 1. Tables are presented with a descending order of Response % for each characteristic.

## Results

### Participants information

Forty-one free-range egg farmers were identified in the original dataset, with an outdoor stocking density of ≤1500 hens/hectare, and included in this study. The post codes collected as part of the demographic information showed that respondents originated from all states except Northern Territory and Australian Capital Territory, with the majority from Victoria (32%), followed by South Australia (24%), New South Wales (22%), Queensland (12%), Western Australia (7%) and Tasmania (2%). All respondents except two provided their names, location and phone numbers and e-mail contact while 56% farmers provided their farm website. Eighty-three percent of respondents answered the questions online, whilst the remainder indicated a phone call would be preferred to complete the survey.

### Farm and flock characteristics

Two main types of free-range housing were identified with 56% of the respondents using mobile (modified caravan/trailer) housing, and 44% using fixed sheds. Forty-one percent of respondents regardless of whether they had mobile or fixed sheds had rotational range access in place. Respondents reported indoor stocking densities between 6–11 hens/m^2^ with the median stocking density of 8 hens/m^2^ in the barn/mobile caravan. The total ranging area per farm varied from 0.10 to 5.05 hectares. Eighty-three percent of the respondents operated only one farm. Twenty-four percent of respondents housed <1000 hens in total, while 37% and 39% respondents housed 1000–5000 and >5000 hens respectively. While 39% of respondents housed an average flock size of <1000 hens, 41% respondents had >3000 hens/flock. Fifty-six percent of respondents managed 2–10 flocks per year, indicating continuous production cycles on most farms (Table 2).

**Table 2:** Farm and flock characteristics of respondent free-range egg farms with an outdoor stocking density of ≤1500 hens/hectare.

Characteristics	Options	Count	Response (%)	CI (%)	SE (Prob)
**Number of farms owned**					
	1	34	82.93	68.73–91.47	0.0588
	2–5	4	9.76	3.85–22.54	0.0463
	>5	3	7.32	2.51–19.42	0.0407
**Breed of hen**					
	Isa Brown	18	43.90	29.90–59.00	0.0775
	Hy-line Brown	16	39.02	25.65–54.27	0.0762
	Bond Brown	5	12.20	5.32–25.54	0.0511
	Hisex Brown	1	2.44	0.43–1.25	0.0241
	Lohmann Brown	1	2.44	0.43–1.25	0.0241
**Number of flocks housed/year**					
	2–10	23	56.10	41.04–70.11	0.0775
	<2	14	34.15	21.56–49.45	0.0741
	>10	4	9.76	3.86–22.55	0.0463
**Average flock size (hens)**					
	>3000	17	41.46	27.76–56.63	0.0769
	<1000	16	39.02	25.66–54.27	0.0762
	1000–3000	8	19.51	10.23–34.01	0.0619
**Total number of hens/farm**					
	1001–5000	15	36.59	23.59–51.88	0.0752
	10001–50000	12	29.27	17.61–44.48	0.0711
	≤1000	10	24.39	13.83–39.34	0.0671
	>50000	2	4.88	1.35–16.14	0.0336
	5001–10000	2	4.88	1.35–16.14	0.0336

Two breeds of hens were predominantly used, ISA Brown (44%) and Hy-line Brown (39%) (Table 2). Approximately 27% respondents used other breeds such as Bond Brown, Bond White, Bond Black, Lohmann Brown, Plymouth Rock and Australorp, with one farmer breeding their own hybrid layers for free-range production.

Of the farms with fixed sheds, 83% used wood shavings and straw as their bedding material, while the rest had a raised floor with plastic slats.

#### The range and its components

Fifty-one percent of the respondents had sown pasture on the range and 46% had planted shrubs or trees, while 12% respondents reported native growth of local species including various herbs and existing remnant or native pastures. Some of the most common plant species found on the range varied from Rye grass (*Lolium* species), Kikuyu (*Pennisetum clandestinum*), Fat hen (*Chenopodium album*), Stinging nettle (*Urtica dioica*), Marshmallow (*Malva parviflora*), Amaranth (*Amaranthus* species), Poke weed (*Phytolacca* species), Clovers (*Trifolium* species), Cocksfoot (*Dactylis glomerata*), Rhodes grass (*Chloris gayana*), Buffalo grass (*Stenotaphrum secundatum*) and Couch grass (*Cynodon dactylon*), Lucerne (*Medicago sativa*), native grasses (various species) and African love grass (*Eragrostis curvula*). On some farms, remnants of crop or vegetable harvest were utilised and included Oats (*Avena sativa*), Sorghum (*Sorghum bicolor*) and pulse crops such as Faba beans (*Vicia faba*). Bushes and trees like Wattle (*Acacia* species) and Eucalypt/gum (*Eucalyptus* species), Almond (*Prunus dulcis*), Olive (*Olea europaea*), Pine (*Pinus* species) and Tea Tree (*Melaleuca* species), Comfrey (*Symphytum officinale*), Wormwood (*Artemisia absinthium*) and Rosemary (*Rosemarinus officinalis*) were also reported to be present on the range.

Eighty-two percent respondents did not re-sow and 83% respondents did not irrigate their range areas. Respondents reported the soil on their range to be: loam (34%), clay (24%), sandy loam (20%), sandy (15%) and light clay (15%) while others reported mixed types that included heavy black soil and cracking clay.

**Fig 1:**
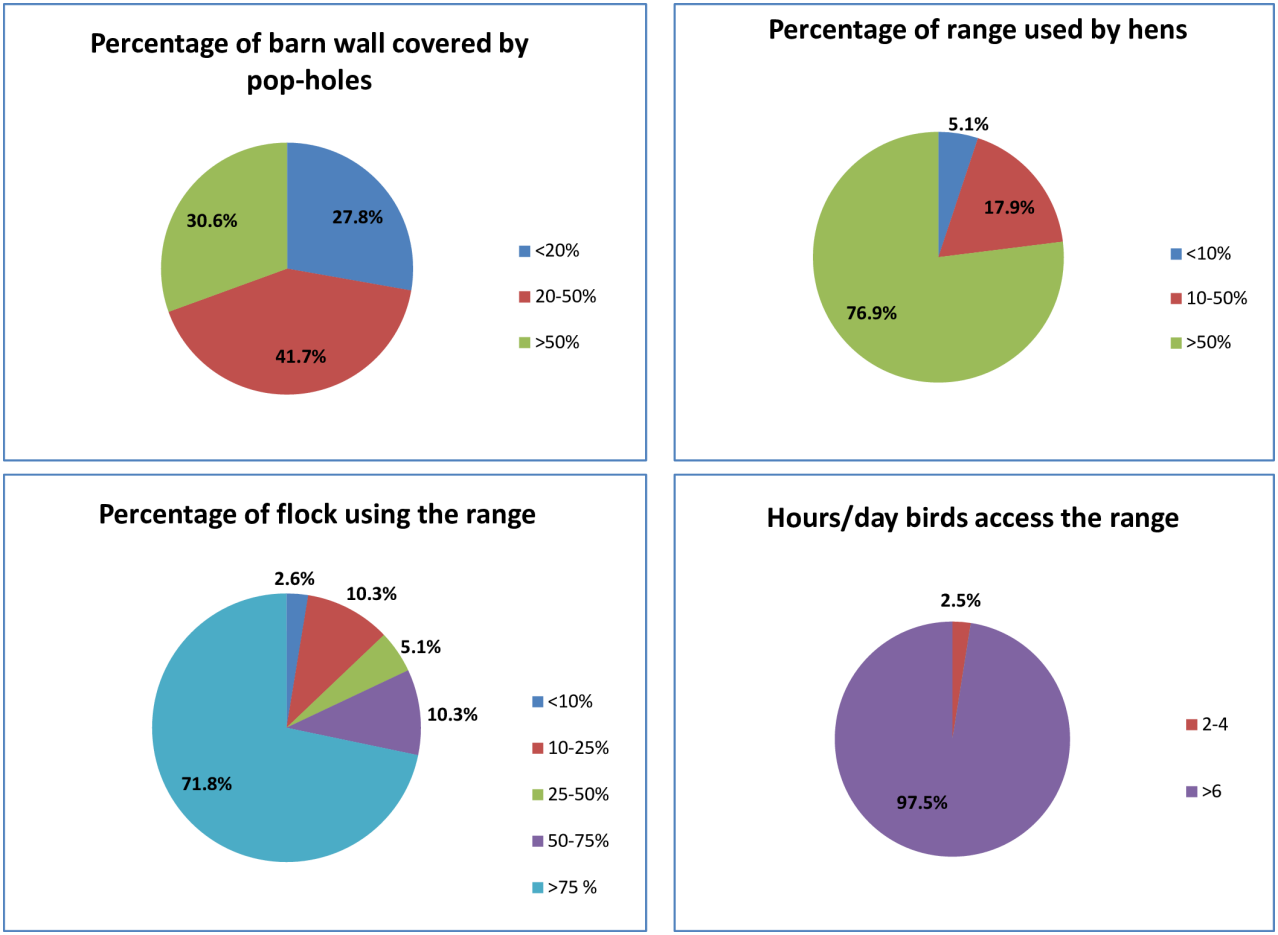
Range access on respondent free-range farms with a stocking density of 1500 hens/hectare. (a) Percentage of barn wall covered by pop-holes (b) Percentage of range used by hens (c) Percentage of flock using the range area (d) Hours/day birds access the range.

Access to the range was determined by asking respondents to comment on the percentage of their barn walls being covered by pop holes. Forty-one percent of respondents reported 20–50% of the barn walls were covered with pop-holes, while 34% respondents stated that more than 50% of the barn wall allowed for access to range. Seventy-two percent of the respondents reported that more than 75% of the birds in a flock used the range area. Seventy-seven percent respondents reported >50% of the range area to be used by the birds. The majority (98%) of respondents reported that birds accessed the range for more than six hours on any given day (Fig 1).

#### Climatic conditions

The climate of a region determines the setup of the outdoor range areas. Table 3 describes the climatic conditions in the year preceding this survey for the forty-one respondent egg farms included in this study. The average annual rainfall was >600 mm on 48% farms with 7% reporting an average annual rainfall of <200 mm. Fifteen percent of the respondents reported maximum temperature of >40°C with another 22% reporting maximum temperature of 30–40°C. Sixty four percent of farmers reported less than favourable minimum temperature of <10°C.

**Table 3:** Climatic conditions in the preceding year on respondent farms.

Characteristics	Options	Count	Response (%)	CI (%)	SE (Prob)
**Average Rainfall**					
	400-600mm	13	32	19.36–46.98	0.0726
	>800mm	10	24	13.82–39.34	0.0670
	600-800mm	10	24	13.82–39.34	0.0670
	200-400mm	5	12	5.32–20.55	0.0511
	<200mm	3	7	2.51–12.42	0.0246
**Average maximum temperature**					
	20–30°C	22	54	38.74–67.94	0.0778
	30–40°C	9	22	12.00–36.70	0.0646
	>40°C	6	15	6.88–21.40	0.0552
	<20°C	4	10	3.85–22.54	0.0463
**Average minimum temperature**					
	0–10°C	21	51	36.48–65.74	0.0780
	10–20°C	15	37	23.58–51.87	0.0752
	<0°C	5	12	5.32–20.55	0.0511
**Extreme weather conditions**					
	Drought	14	34	21.55–49.45	0.0740
	Severe storms	11	27	15.69–41.93	0.0692
	Bushfires	8	20	10.23–34.01	0.0681
	Flood	8	20	10.23–34.01	0.0681
	Tropical cyclone	2	5	1.34–16.1	0.0336

In terms of extreme weather conditions, 34% of farmers included in this study had experienced extreme drought conditions, with extended periods (up to a fortnight) of high temperatures (>40°C), and some weather extremes accompanied by bushfires in the year preceding the survey. Other extreme conditions reported by respondents were severe storms, flooding and tropical cyclones (Table 3).

### Housing and feeding

#### Environmental control

Fifty-one percent of respondents did not have any environmental control of their hen houses as they had mobile sheds or vents in the roof of the mobile sheds. However, 15% of respondents reported tunnel ventilation in their sheds. Other environmental control measures included foggers (29%), side curtains (24%) and roof insulation (24%) (Table 4). Eighty percent of the respondents reported trees were the main source of shade on range while 87% reported artificial structures such as shade cloth or constructed shade areas as well as areas under the caravan or solar panels as sources of shade (Table 4).

**Table 4:** Environmental control on respondent farms.

Characteristics	Options	Count	Response (%)	CI (%)	SE (Prob)
**Types of environmental control**					
	None	21	51	36.48–65.74	0.0780
	Foggers	12	29	17.60–44.47	0.0710
	Roof insulation	10	24	13.82–39.34	0.0670
	Side curtains	10	24	13.82–39.34	0.0670
	Tunnel ventilation	6	15	6.38–28.40	0.0552
**Source of shade on range**					
	Trees/ bushes	33	80	65.98–89.76	0.0618
	Shade cloth	16	39	25.65–54.27	0.0761
	Constructed shade	17	41	27.75–56.63	0.0769
	Winter gardens	3	7	2.51–19.42	0.0406

#### Feeders and drinkers

Feeding systems predominantly consisted of feed hoppers (32%) and pan feeders (automatic/gravity refill) (28%), while chain feeders and feed troughs were used by 20% and 17% respondent farmers respectively. Seventy-eight percent of the respondents provided feed *ad libitum*. The feeders were predominantly located in the barn (54%) but a large number of farmers placed them on the range (32%) or on both the range and barn (15%) (Table 5).

**Table 5:** Feed characteristics and management as reported by respondent farms.

Characteristics	Options	Count	Response (%)	CI (%)	SE (Prob)
**Feeding frequency**					
	*Ad libitum*	32	78	63.25–87.99	0.0646
	2–5 times	5	12	5.32–25.54	0.0511
	<2 times	2	5	1.34–16.13	0.0336
	>5 times	2	5	1.34–16.13	0.0336
**Location of feeders**					
	In the barn	22	54	38.74–67.94	0.0778
	On the range	13	32	19.56–46.98	0.0726
	Both in the barn and on the range	6	15	6.88–28.44	0.0552
**Feed manufacturing**					
	Milling facility	30	73	58.06–84.30	0.0692
	Own production	9	22	12.00–36.70	0.0646
	Both of the above	2	5	1.34–16.13	0.0336
**Physical form of the diet**					
	Complete diet	32	78	63.29–87.99	0.0646
	Combined feeding	6	15	6.88–28.44	0.0552
	Choice feeding	3	7	2.51–19.42	0.0406
**Structure of feed**					
	Pellet	11	27	15.69–41.93	0.0692
	Coarse ground mash	11	27	15.69–41.93	0.0692
	Fine ground mash	7	17	8.52–31.26	0.0587
	Whole grain with mash or pellet	6	15	6.88–28.44	0.0552
	Other	6	15	6.88–28.44	0.0552
**Additional Sources**					
	Shell grit	17	41	27.75–56.63	0.0769
	Limestone particles	15	37	23.58–51.87	0.0752
	Hay	11	27	15.69–41.93	0.0692
	Silage	3	7	2.51–19.42	0.0406

Drinking systems consisted of nipple-cup drinkers installed in 54% of the housing facilities while open water sources such as bell and trough drinkers accounted for the remaining 46%.

#### Feed characteristics

Seventy-three percent of the farmers included in this study sourced their feed from a milling facility. A complete diet was provided for hens on 78% of the farms. The form of the feed varied from coarse ground mash (27%) to fine ground mash (17%), whole grain with mash or pellets (15%), pellets (27%) and others (15%) which included extruded and cooked soy, crumble and kibble (Table 5). Additional sources of feed included shell grit (41%), limestone particles (37%), hay (27%), silage (7%) (Table 5) and others including marble chip, barley, straw, pasture, seaweed meal and diatomaceous earth. All respondents reported range component ingestion by hens in the form of vegetation, insects or stones and grit. Feed intake was reported to vary from 80–115 g/hen/day at placement to 105–120 g/hen/day at peak of lay.

#### Nesting

Twenty-seven percent of respondents reported having rollaway nest boxes with manual collection while 39% reported automatic rollaway nests. Wooden boxes with manual collection of eggs (24%) and plastic drum cut-outs (10%) were the other common methods of egg collection.

### Rearing and production

#### Pullet rearing

Majority of the respondent farmers (63%) sourced their pullets from rearing facilities at the age of 12–17 weeks, while 37% of farmers sourced them as day-olds and reared the hens themselves. Only 5% farmers hatched pullets on farm. Forty-four percent of the respondent farms reported that the pullets they received had been reared on barns while 44% reported them to have been reared on barns with access to outdoor range. However, 12% of respondents did not know how the pullets had been reared before they arrived on the farm. The distance between the rearing facility and the laying farm varied from 3 km to 600 km.

#### Laying hen performance

The layers on respondent farms were kept in lay for an average of 83 weeks (ranging between 63–104 weeks). Thirty-six percent of farmers did not record their hen’s laying performance. For the farms that did record, the average laying performance was reported to be 75% and varied between farms from as low as 40% to 97%. Recording of bird weights was reported by 52% of farmers included in this study. The average body weight for hens that were placed at 17–19 weeks of age varied from 1300 to 1600 g while the average weight of hens at peak lay averaged between 1800 to 2100 g. Uniformity of hen weights for the flock at placement and peak of lay, ranged from 60–90% and 80–95% respectively.

### Health and welfare

Less than 5% mortality was reported for pullets by 68% of respondents and for hens at peak of lay by 62% respondents, while the remaining farmers reported up to 10% mortality. Sixty-four percent of the respondents purchased hens that were not beak trimmed. Of the ones that did purchase beak trimmed hens, 90% were treated using the infrared method at day-old. The major causes for mortality were reported to be predation (34%), cannibalism (29%), grass impaction (19.5%), heat stress (24%) and disease outbreaks (10%). Other reasons listed for mortality included cold temperatures at night, smothering, vent prolapse, old age and injury. The most prevalent transmittable diseases seen in the hens were fowl cholera (17%) followed by coccidiosis (7.3%), spotty liver (7.3%) and infectious laryngotracheitis (ILT) (4.8%). Other diseases included infectious bronchitis (IB) and fowl pox. Thirty-two percent of respondents reported their hens to be affected by both internal and external parasites. The remaining 68% of respondents had either never checked or could not see any signs of parasites. Up to 50% of the respondents were neither satisfied with the control options for preventing and treating internal and external parasites, nor the options to treat or control viral and bacterial diseases. Seventy percent of respondents reported no regular veterinarian visits to the farm but contacted one as and when required.

### Biosecurity

Eighty percent of respondents had fenced the range areas. A copy of the “National Farm Biosecurity Manual-Poultry Production” [18] was kept on 49% respondent farms (Table 6). Cleanup of feed spills, mowing of grass around production area, disinfectant trays and protective clothing and footwear, were some of the biosecurity measures implemented by 40–50% of the respondents. Chlorination of water, fly and beetle control and appropriate storage of litter and manure were practiced by only 10–20% of respondents (Table 6). Other biosecurity measures that some farmers followed (not listed in Table 6) included rodent control, restriction of visitors and rotation to fresh pasture regularly. Two farmers did not use any biosecurity measures.

**Table 6:** Biosecurity measures as implemented on respondent farms.

Biosecurity Measure	Count	Response (%)	CI (%)	SE (Prob)
Fenced off range areas	33	80	65.98–89.76	0.0618
Feed spills cleaned up as soon as possible	21	51	36.48–65.74	0.0780
A copy of 'National Farm Biosecurity Manual' available on farm	20	49	34.25–63.51	0.0780
Grass on and around production area is regularly mowed	19	46	32.05–61.25	0.0778
Disinfectant trays on entrance to sheds	17	41	27.75–56.63	0.0769
Wearing protective clothing and footwear while entering the shed	16	39	25.65–54.27	0.0761
Chlorination of water	10	24	13.82–39.34	0.0670
Fly and beetle control	7	17	8.52–31.26	0.0587
Litter and manure appropriately stored after final pick up in meat chickens	5	12	5.32–25.54	0.0511
Other (please specify)	10	24	13.82–39.34	0.0670

### Manure management

#### Vegetative cover and water bodies

Sixty-six percent of farms had a gentle to moderate slope on the range whilst 32% reported a flat range area. Uniform or moderately variable vegetative cover was maintained in most seasons on 41% and 37% respondent farms respectively, while 5% of respondents had little or no cover most of the time. Eighty-eight percent of respondents rotated range areas to maintain the vegetative cover.

Thirty-seven percent of farms had drainage lines running through the range area. Fifty-four percent of the respondents had a watercourse or an on-farm open water storage body within 500 m from the range. Sixty percent of farms had groundwater less than 10 m deep.

#### Manure and surface run-off management

The main areas of manure deposition coincided with where the hens congregated, i.e. under shade structures and trees (Table 7). Twenty-four percent farmers reported manure deposition inside sheds/barn, while 17% farmers reported manure to be spread over the entire range (Table 7). Fifty-five percent of the farmers that responded to this question did not collect or scrape manure from the highly loaded areas. Of those that collected manure, management involved applying the manure to crop or pasture on-farm (44%), composting on farm (17%), or stockpiling on farm (15%) while 20% farmers distributed the manure to off-site users. Seventy-three percent of respondents did not collect or treat the run-off on their farm, while others used vegetative strips or terminal ponds to collect the waste (Table 7).

**Table 7:** Manure and run-off management on respondent farms.

Characteristics	Options	Count	Response (%)	CI (%)	SE (Prob)
**Main areas of manure deposition**					
	Shade areas	11	27	15.69–41.93	0.0692
	Inside sheds and barns	10	24	13.82–39.34	0.0670
	Near shelter vegetation	9	22	12.00–36.70	0.0646
	All over the range	7	17	8.52–31.26	0.0587
	Below mobile units and caravans	6	15	6.88–28.44	0.0552
	Outside pop holes	6	15	6.88–28.44	0.0552
**Management of collected manure**					
	Applied to crop or pasture on-farm	18	44	29.89–58.95	0.0775
	Sold (or given) to off-site users	8	20	10.23–34.01	0.0681
	Composted on-farm	7	17	8.52–31.26	0.0587
	Stockpiled on-farm	6	15	6.88–28.44	0.0552
	Other	2	5	1.34–16.13	0.0336
**Management of run-off**					
	Run-off not collected or treated	29	73	57.16–83.89	0.0784
	Run-off treated in a vegetative filter strip	9	22	12.00–36.70	0.0646
	Run-off collected in a terminal pond and irrigated onto crop or pasture	2	5	1.34–16.13	0.0336

Soil nutrient monitoring of the range was regularly undertaken by 24% of the respondents and 17% monitored surface water quality including nearby water bodies and dams on farm. Seven percent of respondents monitored groundwater quality using bores or piezometers, while one farm, which practiced co-grazing with cattle, undertook nutrient monitoring of pasture samples and cattle dung. Odour was not reported to be of concern on any of the respondents’ farms.

### Factors influencing establishment of a free-range production system

**Fig 2:**
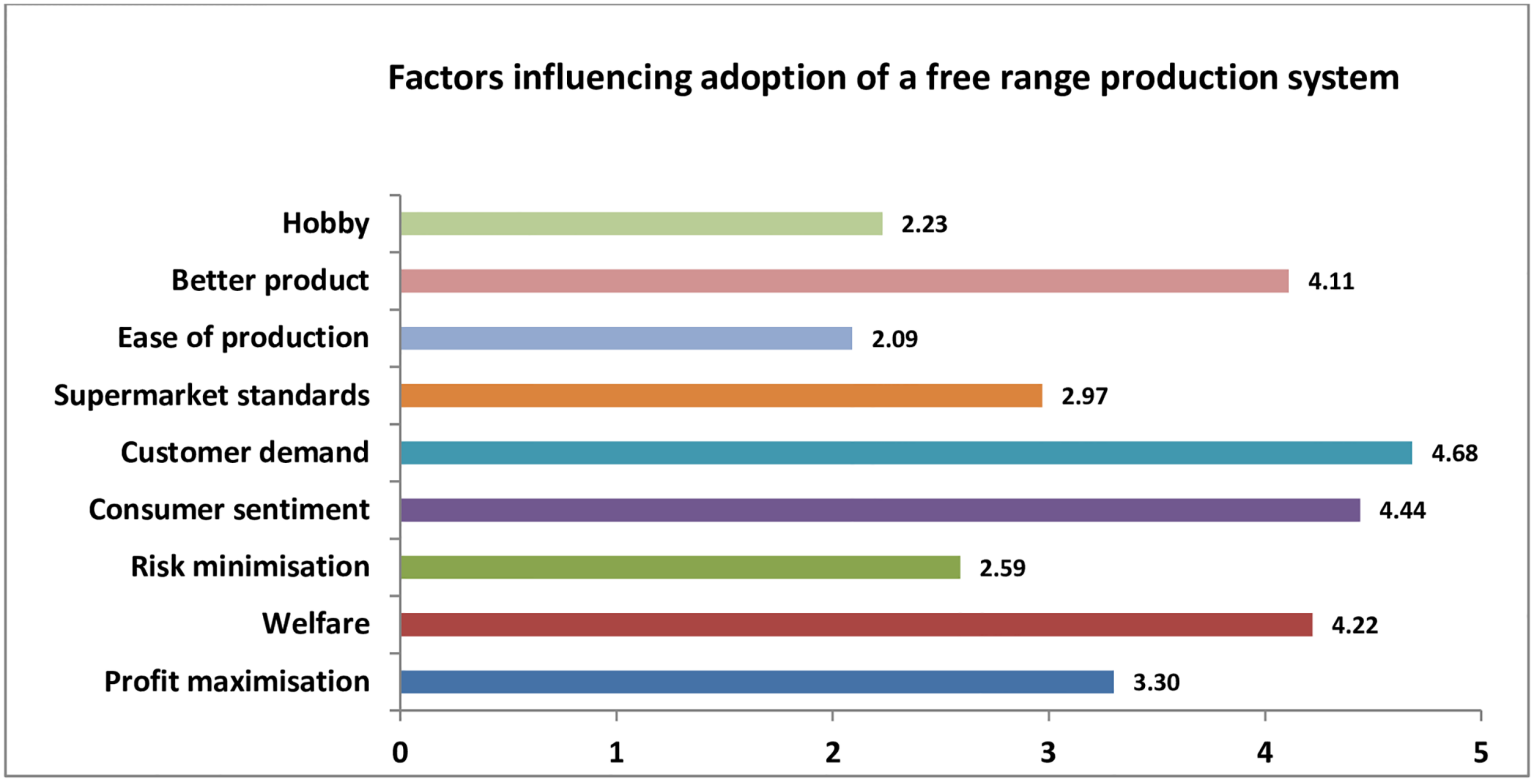
Factors influencing adoption of free-range production system on respondent farms. The scale from 0–5 denotes the weighted averages across the five categories of agreement (Strongly disagree, Disagree, Neither agree nor disagree, Agree and Strongly agree).

Customer demand, consumer sentiment and welfare were rated as the major factors for influencing farmers to establish a free-range egg farm, followed by their belief that the free-range systems resulted in a better product (Fig 2).

Eighty-three percent of farmers indicated they would prefer to house all their hens in the free-range system, while 16% would prefer to house at least 50% of their hens in a conventional cage system to be able to sustain and make a profit. Forty-four percent of the respondents anticipate operating their free-range enterprise for more than 20 years, while 10% were looking at a short-term venture of less than 5 years. The major support system for consultation and decision making about their enterprise was reported to be other free-range farmers (44%), independent consultants (32%), researchers (20%), and supermarkets (10%). Support was also sought from veterinarians, nutritionists and other family members.

Ninety percent of the farmers indicated the need to acquire moderate to high level of new skills and knowledge to start a free-range enterprise with all reporting a lack of appropriate training, education material and personnel to answer their numerous queries. A majority of farmers (83%) felt that there was no sufficient scientifically validated knowledge available, relevant to Australian conditions, to assist producers in establishing successful free-range production enterprises. Accreditation to a free-range organisation had been gained by 54% of farmers with 20% considering obtaining it.

Pasture management, nutrition and health were the areas of highest interest that free-range egg producers wanted researched, followed by welfare, environmental impact, animal housing and economics (Table 8). Other areas that free-range farmers would like to invest in research were education and training for farm owners, managers and staff, mobile and rotation ranging, egg quality at end of lay, control of sparrows and wild birds, floor eggs, reducing labour costs and regulations on advertising and labeling.

**Table 8:** Research areas of interest to respondent farms.

Area of research	Count	Response (%)	CI (%)	SE (Prob)
Pasture management	24	59	43.36–72.24	0.0769
Nutrition	24	59	43.36–72.24	0.0769
Health	24	59	43.36–72.24	0.0769
Welfare	22	54	38.74–67.94	0.0778
Environmental impact	20	49	34.25–63.51	0.0780
Animal housing	18	44	29.89–58.95	0.0775
Economics	10	24	13.82–39.34	0.0670

## Discussion

The present study included forty-one free-range egg farms, all with a stocking density of ≤1500 hens/hectare and was able to document baseline information on demographics, farm and flock characteristics, outdoor range and range access, housing, nutrition, production parameters, disease incidence, causes of mortality, biosecurity practices and environmental impact. The information generated in this study enables a deep understanding of the numerous semi-intensive free-range systems, which are widely interspersed in Australia but have not been documented to date.

The consumer perception of free-range farms involves an idyllic scenario with portable laying sheds, where hens are out on the paddock all day, scratch the soil for insects and seeds, and consume green pasture, while displaying natural behaviours such as dust bathing. Maremma dogs or alpacas guard these birds and feed and water is provided on the range. It is this perception by consumers of hen welfare and the extra labour required to manage these farms that drives the higher prices for free-range eggs (average grocery egg price of AUD $5.40 (per dozen for free-range eggs as compared to $3.24 per dozen for cage eggs [5].

Two types of housing systems were identified to be prevailing under stocking density of ≤1500 hens/hectare in this study: mobile housing and fixed sheds. Of the fixed sheds, some facilities are converted broiler sheds or conventional cage layer sheds while others are a simple shed with minimal or no insulation, open sidewalls, with pan feeders and nipple-cup drinkers. The caravan or trailer style mobile sheds are purpose-built for free-range layers and can house up to 2000 hens. These are roadworthy and can be moved every few days around the paddock to evenly distribute manure and provide the chickens with fresh areas to graze on. The caravan style sheds, can have manual or rollaway nest system, in some cases with a conveyer belt egg collection on one end of the caravan. Feed and water are either provided on range or can be inbuilt in some modern purpose built mobile sheds. Usually an electric fence and Maremma dogs or Alpacas are used to discourage predators. However, there is no uniformity in these types of sheds or mobile units with each farmer adapting the system to what suits their farm demographics and husbandry practices. Most of these farms sell eggs at the farm gate, farmers’ markets or to regional stores [8].

Forty-seven percent of the farms in this study were not accredited to any free-range organisation. For those farms that were affiliated to Free-range Egg and Poultry Australia Ltd. (FREPA), the indoor stocking density of FREPA guidelines was determined by the flock size which could not exceed 10 hens/m^2^ when up to 1000 hens/flock are housed, 9 hens/ m^2^ when up to 2000 hens are housed, and 8 birds/m^2^ when up to 3000 hens were housed [4]. However, in order to determine the outdoor stocking density, FREPA refers to the Australian Model Code of Practice which states the maximum stocking density for the outdoor area is 1500 hens/hectare (6.6 m^2^/hen) [2]. Similar outdoor stocking density is also referred to in the standards from the Royal Society for the Prevention of Cruelty to Animals [3] and The Australian Certified Organic Standards [19].

The predominant breeds of hens, used on participant farms, include Isa Brown and Hy-line Brown. Both these commercial brown laying hen breeds have a European origin and have been genetically selected for improved productivity and feed conversion ratio for intensive in-house cage production systems. Furthermore, current feed recommendations have also been developed based on housing in a climate-controlled environment with limited hen movement [20]. These circumstances may contribute to the fact that average hen body weight / flock varied from 1.42 kg-2.2 kg and uniformity of the flock ranged from 50–96% on farms included in this study. There has been a recent move into developing a breed for Australian conditions that have been selected for free-range characteristics such as socializing, foraging, ranging and stress and disease resistance.

The egg producers in Australia are distributed across a large geographical area, with highly diverse climatic conditions. Extreme weather conditions were reported by respondents experiencing temperatures of >30°C and <10°C at certain times of the year. Furthermore, some farms were exposed to extreme droughts, heat waves, and bushfires. Despite this, 51% of free-range egg producers reported having no environmental control in their layer housing systems. Lack of environmental control can lead to high temperatures in the sheds during hot summer months, leading to lowered feed intake. In addition to this, hot water in drinking pipes and inadequate shade on range can lead to excessive panting resulting in respiratory alkalosis, and ultimately heat stress in hens. Twenty-four percent respondents indicated heat stress as a cause of mortality on their farm. Hens are able to cope better and have an improved feed intake when they are allowed to feed during the cooler part of the day and if they have access to cool water [21, 22]. This becomes very important in mobile shed situations where the hens are invariably forced to face the harsh weather conditions, as climate control is not available.

Nesting facilities were sub-optimal on respondent farms with nearly 34% reporting either wooden boxes or plastic drum cutouts as nests. The provision of suitable nest boxes to enable hens to show normal pre-laying and nesting behaviour is a priority, particularly as oviposition approaches. Moreover, in the absence of suitable nest-boxes, hens tend to move away from other hens in the flock and find a secluded place on the range in which to nest on the ground. From a management perspective, this is a costly choice with regards to egg hygiene and food safety concerns, as eggs may be predated, damaged, dirty and need to be manually collected. Thus farmers operating free-range systems need to be informed on research involving familiarization of hens to indoor nest boxes and using them before being allowed access to range [18, 19]. The indoor nest boxes need to be sufficiently attractive so that hens are willing to select them in preference to other sites.

Fresh pasture cover and availability of fodder on the range is a requirement mentioned in most accreditation guidelines [2, 4, 19]. For example, the Australian standards for free-range egg production as required by FREPA state that the range area must be capable of continued production of vegetation and that the land where hens are permitted to range must have shade, shelter and palatable vegetation. Australian Certified Organic stipulates that the range for organic certified hens shall include edible forage at all times [19]. A number of plant species have been reported as growing on range areas on farms identified for this study. Responses indicated some variations in sown plant species reflecting the differences in climatic and soil conditions across Australia. Furthermore, there were 35 weed species growing on the range with some found to have a wide geographic distribution across the farms.

More than 75% of birds were reported as using the range and 96% respondents reported that birds accessed the range for more than six hours on any given day. Free-range laying hens in this study have been reported to spend up to 75% of their day outdoors. A potential feature of access to outdoor areas is the availability of supplementary feed items, whether animal, vegetable or mineral [23–27]. All farmers indicated ingestion of range components in this study. However, quantification of range components is difficult. Although one of the requirements of FREPA is for free-range poultry to have access to range area that is ‘mainly covered with vegetation’, the nature of the vegetation is not specified. The pastures need to be evaluated for their nutritive value and intake by the hens quantified in order to make adjustments to feed formulations. Information on quality and biodiversity of the range area, stocking density, and behavioural factors such as the willingness and ability of the birds to move over the range area and select from its resources becomes essential to free-range management.

Nutrient requirements of a standard brown egg laying hens focus on 100–120 g feed intake/hen/day, but these estimates may not be suitable for free-range birds who display increased energy requirements because of additional physical activities and the need for temperature maintenance [28]. In commercial flocks, the range may be used by 5–95% of the hens [29, 30]. A previous study revealed that, when housed in fixed sheds, up to 10% of layers never leave the hen house, while others spend a variable time on the range [31]. Hence, the freedom of choice results in the development of several sub-populations within one flock [32]. The need for alternative feeding strategies is reflected in on-farm practices, which include feed supplementation with shell grit, limestone, hay, silage, and others including vegetables, pasture, insects, and harvested grass.

Excessive pasture intake can result in reduced consumption of a balanced diet, leading to reduced intake of energy and essential nutrients such as amino acids, and leading to malnutrition and severe loss of body condition in sub-clinical cases, and death in severe cases. Range usage can also frequently be associated with intestinal grass impaction [17, 33]. Nearly 19.5% of respondents indicated that grass impaction to be a cause for mortality. In order to minimise the intake of excessive fibre materials such as long grass, range management such as mowing or co-grazing with cattle or sheep should be considered.

The focus on hen welfare is reflected in concern about severe feather pecking and cannibalism and the various methods of beak trimming [20, 34]. Based on information provided by the respondents, half of the free-range farms did not beak-trim their hens. Both the Australian Model Code of Practice and the FREPA standards support minimal beak trimming by competent persons qualified under the national competency standards [2, 4]. Therefore, the practice of infrared trimming at day one and additional hot blade trimming later in life is common in Australian laying hens. However, some certifying organisations do not allow for beak-trimming, which could be attributed to occurrence of aggression, severe feather pecking, and cannibalism on some free-range farms.

Proactive health management and good biosecurity is important in free-range poultry production especially due to the restriction on use of in-feed antibiotics. Free-range poultry production has been implicated in the increased likelihood of contact between chickens and wild birds, thus potentially increasing the risk of Avian Influenza introduction and outbreaks [35]. This was highlighted by the fact that 54% of the respondent farms reported the presence of either a watercourse or an open water storage body within 500m of the outdoor range. For standard biosecurity measures, lower levels of execution were found with eight of ten actions implemented by <50% of respondents in this study, providing evidence that improved biosecurity guidelines and implementation is needed for semi-intensive free-range farms in Australia.

Fifty-five percent of respondents did not collect or scrape manure from highly loaded areas and 73% of respondents did not collect or treat run-off on their farms. Ground and surface water pollution can occur through leaching when the nutrient and trace elements in manure get accumulated in the soil. High levels of these nutrients and elements can be toxic to vegetation [36]. In order to minimize adverse environmental impacts, factors such as the type of vegetation species, level of ground cover, stocking rates on range and manure management practices need to be evaluated across a wide range of Australian climatic conditions and soil types and best practices put in place in order to maintain long-term sustainability and social acceptance of free-range production.

Although the discussion of a nationwide definition of “free-range” may have been settled for now, it does not establish any meaningful standards and uniformity for farms with ≤1500 hens/hectare stocking density, thus allowing for these farms to keep increasing in number with different forms and specifications. Customer demand and consumer sentiment are amongst the major factors for farmers to establish free-range production systems. However, a comprehensive and informative platform for first-time and long-term free-range farmers is one of the gaps that exist in the industry. Ninety percent of respondents admitted to having to learn new skills and almost all commented on a lack of related training, education material and personnel for consultation. Although training is available for intensive free-range systems, there is not much offered that is targeted at semi-intensive mobile free-range systems. Moreover, new scientifically validated information created for the intensive free-range sector on a regular basis, is not transferrable to these semi-intensive systems, specifically the mobile systems. This is highlighted by the fact that respondents’ support system for consultation and decision making mainly involved other free-range farmers followed by independent consultants, researchers and nutritionists.

## Conclusion

This study was able to capture a previously undocumented section of the industry comprising of small to medium–scale farms that are not usually affiliated to any industry service body, but have emerged in big numbers across Australia and provide their products either through farmer’s markets or local stores. Although this study has attempted to document the practices, other studies are needed to conduct a census and record all enterprises that fall under this category. It is an important section of the industry and needs to be accounted for, to realise the full scope of egg production in Australia.

Some of the unique characteristics of these semi-intensive free-range farms with an outdoor stocking density of ≤1500 birds/hectare include the presence of two types of housing systems, the fixed sheds and the mobile/caravan style housing. No scientifically validated research has been conducted to optimise the climate control, nesting and feeding in the mobile housing systems and to determine their effects on nutrition, health and welfare of birds. Range usage has been reported to be high in these systems with majority of birds accessing the range area and a large proportion of the outdoor range area being utilised. Dilution of allocated feed rations by elements consumed on range along with extra requirements due to temperature maintenance and increased physical activity need to be taken into account while formulating diets for free-range birds. High mortality rates have been attributed to predation, grass impaction, heat stress, disease and parasites, and smothering, all of which had been eradicated by introduction of intensive systems in the past but have re-emerged in these free-range systems. Adequate veterinarian support and training is a major requirement to manage and contain these conditions. Biosecurity could be improved on the farms with improved uptake of a number of actions by regular monitoring and dedicated workshops relevant to these systems. Management of manure and run-off needs to be put in place to prevent pollution of ground and surface water through leaching. Better legislation on planning of the farms is needed, to provide sustainable and environmentally conscious systems which account for welfare of birds. Overall a nation-wide training and support network consisting of experts on production and management, nutrition, health, welfare, environmental impact and biosecurity can help to sustain and expand these systems on a long term basis. Data and traceability for these farms is important to account for the total egg production statistics and to ensure health and welfare for all types of free-range enterprises in Australia.

## Supporting information

S1 Dataset Minimal dataset. (XLSX)
